# Editorial: Neuroinflammation and distribution of proinflammatory markers in the brain

**DOI:** 10.3389/fnagi.2025.1723530

**Published:** 2025-10-27

**Authors:** Huazheng Liang, Yong Hao, Yong Bi

**Affiliations:** ^1^Shanghai Key Laboratory of Anesthesiology and Brain Functional Modulation, Shanghai, China; ^2^Clinical Research Center for Anesthesiology and Perioperative Medicine, Shanghai Fourth People's Hospital, School of Medicine, Tongji University, Shanghai, China; ^3^Translational Research Institute of Brain and Brain-Like Intelligence, Shanghai Fourth People's Hospital, School of Medicine, Tongji University, Shanghai, China; ^4^Southeast University-Monash University Joint Graduate School, Suzhou, Jiangsu, China; ^5^Suzhou Industrial Park Monash Research Institute of Science and Technology, Suzhou, Jiangsu, China; ^6^Southeast University Joint Research Institute, Monash University, Suzhou, Jiangsu, China; ^7^Department of Neurology, Renji Hospital Affiliated to Shanghai Jiaotong University, Shanghai, China; ^8^Department of Neurology, Zhoupu Hospital Affiliated to Shanghai University of Medicine & Health Sciences, Shanghai, China

**Keywords:** neuroinflammation, neurodegeneration, natural products, therapeutics, ferroptosis, postoperative delirium, ischemic stroke

Neuroinflammation is the immune response within the central nervous system, primarily mediated by activated glial cells like microglia (Gao et al., 2023) and astrocytes (Patani et al., 2023). This response involves the release of pro-inflammatory cytokines, chemokines, and reactive oxygen species (Chen et al., 2024; Goleij et al., 2025). While initially a protective mechanism to restore homeostasis of the central nervous system (Theus, 2024), chronic or dysregulated neuroinflammation becomes detrimental (Adamu et al., 2024). Consequently, persistent neuroinflammation leads to significant neuronal injury and synaptic loss, which is a hallmark of many neurodegenerative diseases (Shi and Yong, 2025). In Alzheimer's disease (AD), it accelerates amyloid-beta (Aβ) and tau pathology (Povala et al., 2025). This self-perpetuating cycle of inflammation and neurodegeneration is a key driver of disease progression. Treatment strategies are increasingly targeting neuroinflammatory pathways. Investigational approaches include the use of non-steroidal anti-inflammatory drugs, biologics to inhibit specific cytokines like tumor necrosis factor alpha (TNF-α), and agents to modulate microglial activation (Thakur et al., 2023). Promoting a shift of microglia from a pro-inflammatory (M1) to an anti-inflammatory (M2) state is a major therapeutic goal for slowing disease advancement. Therefore, discovering new therapeutics to target neuroinflammation is a promising research direction. As AD has a long prodromal period, finding a biomarker to detect the presence of neurodegeneration or neural injury at the early stage is under great demand. This Research Topic, therefore, aimed to collect articles on investigating early biomarkers for neural injury and potential therapeutics for neuroinflammation.

In AD, Aβ plaques and tau tangles activate microglia, sustaining a harmful inflammatory cycle that exacerbates synaptic loss and cognitive decline. In the study by Chen et al., the therapeutic potential of injectable omega-3 fatty acids in a mouse model of AD (5xFAD) was evaluated. A fish-oil-based triglyceride emulsion was administered to pregnant 5xFAD mice during a critical perinatal window of brain development. In young offsprings, this perinatal omega-3 fatty acids suppressed the activation of glial cells (markers like Iba1, GFAP, Trem2) and showed a trend toward reducing pro-inflammatory cytokines. These anti-inflammatory effects were sustained into adulthood, with treated 4–5-month-old mice showing significantly reduced expression of inflammatory molecules like interleukin 6 (IL-6) and TNF-α. Behaviorally, treated pups exhibited increased ultrasonic vocalizations, suggesting improved communication, while adult mice showed enhanced spatial memory in Y-maze tests. Lipidomic analysis of brain tissue revealed that omega-3 fatty acids increased key membrane phospholipids and significantly decreased levels of pro-inflammatory sphingolipids, particularly lactosylceramide which is linked to neurodegeneration. Notably, omega-3 fatty acids did not affect Aβ plaque deposition. Therefore, strategic administration of omega-3 fatty acids can durably modulate neuroinflammation and brain lipid composition, improving cognitive functions independent of Aβ pathology, offering a promising complementary therapeutic approach for AD.

In ischemic stroke, robust neuroinflammation significantly contributes to secondary brain injury. One of the mechanisms is ferroptosis, an iron-dependent form of regulated cell death. In the study by Wu et al., RNA sequencing was performed on a mouse model of middle cerebral artery occlusion (MCAO) and identified 127 differentially expressed ferroptosis-related genes. Through protein-protein interaction analysis and a random forest algorithm, 10 hub genes were identified. Subsequent validation with RT-qPCR confirmed that 9 of these genes were significantly dysregulated in the MCAO model compared to sham-operated mice. Crucially, when acupuncture treatment was applied to the model, the expression of five key genes, including FTH1, SLC40A1, NRAS, CD82, and PTPN18, was significantly upregulated, suggesting that acupuncture may exert its therapeutic effects in ischemic stroke by modulating these ferroptosis-related targets. It was concluded that these genes and pathways are central to ferroptosis in ischemic stroke and represent promising targets for acupuncture's anti-ferroptotic action.

Neurodegenerative diseases are hard to treat due to the long prodromal period and it remains a challenge to diagnose them early. In the study by Kong et al., the association between the serum potassium level and the serum neurofilament light chain (sNfL) was examined with an aim to utilize the serum potassium and sNfL as early diagnostic biomarkers. Their analysis on 1,670 participants from the National Health and Nutrition Examination Survey (NHANES) (2013–2014) revealed a significant positive association between serum potassium and sNfL. After adjusting for numerous potential confounders, individuals with higher serum potassium concentrations had higher sNfL levels. A nonlinear, positive dose-response relationship was observed, and subgroup analyses indicated that this association was stronger in low-to-middle-income groups, non-drinkers, physically inactive individuals, and those with hypertension or diabetes. These suggest that elevated serum potassium concentration may be linked to increased axonal degeneration. The authors propose that hyperkalemia could contribute to neural injury through mechanisms like neuronal hyperexcitability, cytoskeletal instability, neuroinflammation, and oxidative stress, highlighting the potential utility of the serum potassium as part of the clinical assessment of the risk for axonal degeneration-related neurological disorders.

Postoperative delirium (POD) is a common condition present in post-surgery patients, especially in elderlies who undergo major surgeries. Neuroinflammation observed in these patients may contribute to the blood brain barrier disruption and subsequent neural injury. In the mini-review by Leng et al., emerging biomarkers for POD were described in detail, focusing on the intersection of neuroinflammation and neurodegeneration. Key findings indicate that biomarkers related to AD pathology, such as Aβ and hyperphosphorylated tau (pTau181, pTau217), often show associations with increased POD risk, especially in larger cohorts like PNDABLE, though results vary in smaller studies. Markers of neuronal injury, including total tau and the neurofilament light chain, are also frequently elevated in POD patients. The glial fibrillary acidic protein, reflecting astrocyte activation, presents mixed results, potentially due to cohort and assay heterogeneity. IL-6 is highlighted as a critical inflammatory mediator, with both systemic and central roles in POD pathogenesis. Additionally, blood-brain barrier dysfunction markers, such as the CSF-to-plasma albumin ratio, are increasingly linked to POD. The review underscores the need for larger, standardized studies to validate these biomarkers and the need to develop integrated, multimodal tools for predicting and managing POD in vulnerable surgical populations.

Taking these studies together, this Research Topic has provided experimental evidence on the therapeutic effect of omega-3 fatty acids on AD, which is achieved through modulating neuroinflammation in a 5xFAD model. Acupuncture regulates ferroptosis related genes in the MCAO model. The level of patient serum potassium is positively associated with the level of sNfL, suggesting the correlation of patient serum potassium with neural injury. Biomarkers related to neuroinflammation and neural injury have shown their potential in clinical diagnosis. These findings imply that targeting neuroinflammation might offer a new therapeutic approach to manage neurological disorders.
